# SARI prevents ocular angiogenesis and inflammation in mice

**DOI:** 10.1111/jcmm.15096

**Published:** 2020-03-02

**Authors:** Wenqiu Zhang, Lei Dai, Xun Li, Yiming Li, Maurice Keng Hung Yap, Longqian Liu, Hongxin Deng

**Affiliations:** ^1^ Department of Ophthalmology West China Hospital Sichuan University Chengdu China; ^2^ Research Laboratory of Ophthalmology and Vision Sciences West China Hospital Sichuan University Chengdu China; ^3^ Department of Optometry and Visual Science West China Hospital Sichuan University Chengdu China; ^4^ State Key Laboratory of Biotherapy/Collaborative Innovation Center for Biotherapy West China Hospital Sichuan University Chengdu China; ^5^ School of Optometry The Hong Kong Polytechnic University Hong Kong China

**Keywords:** age‐related macular degeneration, choroidal neovascularization, regulated by IFN‐β, Suppressor of AP‐1, Uveitis, vascular endothelial growth factor

## Abstract

SARI (Suppressor of AP‐1, regulated by IFN‐β) is known to play an important role in some systemic disease processes such an inflammatory conditions and cancer. We hypothesize that SARI may also play a role in ocular diseases involving inflammation and neovascularization. To explore our hypothesis, further, we investigated an endotoxin‐induced uveitis (EIU) and experimental argon laser‐induced choroidal neovascularization (CNV) model in SARI wild‐type (SARI^WT^) and SARI‐deficient (SARI^−/−^) mice. Through imaging, morphological and immunohistochemical (IHC) studies, we found that SARI deficiency exacerbated the growth of CNV. More VEGF‐positive cells were presented in the retina of SARI^−/−^ mice with CNV. Compared to SARI^WT^ mice, more inflammatory cells infiltrated the ocular anterior segment and posterior segments in SARI^−/−^ mice with EIU. Collectively, the results point to a potential dual functional role of SARI in inflammatory ocular diseases, suggesting that SARI could be a potential therapy target for ocular inflammation and neovascularization.

## 1 INTRODUCTION

Age‐related macular degeneration (AMD) is a leading cause of irreversible vision loss among elderly people.^1^ It is estimated that, globally, the number of people with AMD would be 196 million in 2020, going up to 288 million by 2040. More than 60% of these cases are in Asia.^2^ Early AMD is characterized by the presence of drusen and retinal pigment epithelium (RPE) pigmentation abnormalities. There are two forms of late AMD: one is atrophic AMD (dry AMD, characterized by atrophy), and the other is exudative AMD (wet AMD, characterized by angiogenesis). Choroidal neovascularization (CNV) in the form of new vessels from the vascular choroid through Bruch's membrane into the retina accounts for most cases of severe visual disturbance because of AMD.^3^ Currently, intraocular injection of vascular endothelial growth factor (VEGF) neutralizing antibody is the first‐line treatment for CNV in wet AMD.^4^ However, some patients fail to respond to the treatment. Thus, there is a need for greater clarity about the pathophysiology process of CNV and more effective treatment strategies.

Intraocular inflammatory disease is a primary cause of vision loss in industrialized countries.^5^ Uveitis, a common inflammatory ocular disease, may encompass the anterior and posterior segments of the eye (eg iris, ciliary body and choroid). Various conditions could lead to uveitis, such as infections, trauma and systemic diseases. The current treatments for uveitis include steroids, macrolides and immunosuppressants,^6^, ^7^ most of which are usually associated with severe complications.^8^ Endotoxin‐induced uveitis (EIU) is a common model which can be generated by systemically injecting lipopolysaccharide (LPS) in an animal eye. It has been widely used to aid understanding of the pathology of human acute uveitis and investigating new pharmacological therapies^9^ and provides a reasonable proxy of the mechanisms underlying HLA‐B27–associated uveitis in humans.

SARI (Suppressor of AP‐1, regulated by INF‐β), also called as BATF2, is a member of BATF (Basic leucine zipper (bZIP) transcription factor, ATF‐like) family, which contains BATF (otherwise known as SFA2), BATF2 and BATF3 (otherwise known as JDP1 and p21SNFT).^10^ Various studies have demonstrated the anti‐tumour role of SARI in multiple cancers, including lung cancer,^11^ prostate cancer,^12^, ^13^ B lymphoma^14^ and colon cancer.^15^, ^16^ Previous studies by us indicated that SARI was down‐regulated in colon cancer^15^, ^17^ and inhibits colon cancer growth through inhibiting the translational activity of HIF‐1α/VEGF and tumour angiogenesis.^15^ SARI is also involved in innate immunity and infection immunity.^18^, ^19^ SARI protects mice from *Mycobacterium tuberculosis* and *Listeria monocytogenes* mediated Type 1 and Type 2 diseases.^18^ During *T cruzi* infection, SARI functions as a negative regulator of IL‐23a in innate immune cells.^19^ We and a previous study by Kayama also demonstrated the protective role of SARI in colitis through regulating macrophage infiltration.^20^

Against this background, we speculated that SARI may have a role to play in preventing angiogenesis and inflammation responses in certain ocular diseases. To explore our speculation further, CNV and EIU models were induced in SARI wild‐type (SARI^WT^) and SARI deficiency (SARI^−/−^) mice. The present study expands the understanding of SARI function and provides a novel therapy target for certain ocular diseases.

## 2 MATERIALS AND METHODS

### 2.1 Animals

SARI knockout (SARI^−/−^, catalogue no. 019085) and SARI wild‐type (SARI^WT^, catalogue no. 002448) mice, 6 to 8 weeks old, were purchased from the Jackson Laboratory. Experiments were approved by the Animal Ethics Committee of Sichuan University. All animal care, husbandry and experiments were conducted in adherence with institutional guidelines for the use of animals in Sichuan University. Mouse gene type was confirmed via the genotyping protocols supplied by the Jackson Laboratory.

### 2.2 Laser‐induced CNV model

Animals were anaesthetized with ketamine (75 mg/kg) and xylazine (5 mg/kg) by intraperitoneal injection after a drop of 0.2% tropicamide, and 1% phenylephrine (Santen) for pupil dilatation was administered to the right eye. A coverslip was lubricated with 2.5% sodium hyaluronate (Akorn) and applied to the surface of the cornea to facilitate a view of the retina. Five or six CNV lesions surrounding the optic nerve were induced with an argon laser (532 nm wavelength, 100 mW, 50 µm spot size, 0.1 second duration) under a slit‐lamp microscope observation. Laser photocoagulation and rupture of Bruch's membrane were confirmed by the formation of a heat‐induced bubble without affecting the blood vessels. Mice with endophthalmitis were excluded. Each group included at least 30 spots from eight mice.

### 2.3 Fundoscopy and funds fluorescence angiography (FFA)

Mice were deeply anaesthetized by intraperitoneal injection with a mixture of ketamine (75 mg/kg) and xylazine (5 mg/kg). Hyaluronate was used to keep the ocular surface moist. The pupils were dilated using 0.2% tropicamide and 1% phenylephrine (Santen). Retinal pathology was assessed using a Micron Ⅳ fundoscopy system (Phoenix Research Laboratories). FFA was carried out 4 minutes after intraperitoneal injection of 25 mg/mL 150KD FITC conjugated dextran (Sigma‐Aldrich). Digital images of eyes were captured for one minute. Angiograms were graded by two experienced ophthalmologists using a leakage score system (Table 1). The area of CNV lesion was measured in a masked fashion using Image J.

**Table 1:** Criteria of CNV leakage scoring

Signs	Score
No staining, faint hyperfluorescence	0
Staining	1
Moderate staining	2
Strong staining	3

### 2.4 Choroidal flat mount

The entire ocular globes were enucleated and fixed in 4% paraformaldehyde for 1 h. Choroid and retinal flat mounts were obtained by carefully removing the cornea, iris and lenses. The remaining eyecups were subjected to four radial incision towards the optic nerve head for flat mounting. The neurosensory retina was detached by sectioning the optic nerve. The retinal pigment epithelium (RPE)/ choroid/sclera complexes were permeabilized overnight with tris‐buffered saline (0.5% BSA, 0.2% Tween‐20 and 0.1% Triton X‐100). After repeated washes, samples were incubated with a 10 mg/mL solution of 40, 60‐diamidino‐2‐phenylindole (DAPI) (1:500), 1 mg/mL solution of Alexa Fluor 568‐conjugated isolectin B4 (1:100) and 0.2 U/mL solution of Alexa Fluor 488‐conjugated phalloidin (1:100; Invitrogen‐Molecular Probes) overnight at 4℃ in blocking solution. Tissues were mounted onto glass slides and cover‐slipped. Images were captured with Olympus Fluoview FV1000 confocal microscopy at 20× magnification. CNV lesions were identified by their geographic location revealed by the IsolectinB4 (IB4) signal. Image J was used to dissect out the areas of hyperfluorescence for further analysis by blinded observers.

### 2.5 Immunohistochemistry

Eyes were fixed in 4% paraformaldehyde freshly made in PBS overnight at 4°C and cryoprotected in 30% sucrose, and then embedded in paraffin. Retinal sections were sagitally cut through the cornea‐optic nerve axis (3‐μm thick), mounted on slides and dried. After deparaffinization with graded ethanol and xylene solutions, tissue samples were incubated with a blocking reagent, and then with primary antibody against VEGF (Abcam) and CCL 2 (Abcam) at 4°C overnight. After rinsing the slides with PBS, the specimens were treated with secondary antibodies (Zsbio). Secondary Abs were labelled with the HRP, which was detected by diaminobenzidine (DAB; Maixin), whereas the nuclei were stained with haematoxylin (Beyotime). All the sections were examined under an Olympus BX600 microscope and SPOT Flex camera. The VEGF and CCL 2 expressions in each frame were scored as 0, 1, 2 and 3 based on the percentage of positive cells.^21^ Score 0, the percentage of positive cells < 5%; score 1, 5% < the percentage of positive cells < 15%; score 2, 15% < the percentage of positive cells < 25%; score 3, the percentage of positive cells > 25%.

### 2.6 EIU mouse model

Mice were intraperitoneal anaesthetized with ketamine (75 mg/kg) and xylazine (5 mg/kg). EIU was induced in SARI^WT^ or SARI^−/−^ mice by single intravitreal injection of 50 ng lipopolysaccharide (LPS; Sigma‐Aldrich‐Aldrich). The control animals received the same volume of PBS. Animals were divided into 4 experimental groups (Table 2, n = 6 per group). After 24 hours, all animals from each group were killed when the inflammation was provoked and reached its peak.^22^ Eyes were carefully enucleated and processed for evaluation.

**Table 2:** Summary of the treatment groups

Intravitreal injection	Treatment group
2 µL (25 ng/µL) LPS	SARI^WT^ (E)
2 µL (25 ng/µL) LPS	SARI^−/−^ (E)
2 µL PBS	SARI^WT^ (N)
2 µL PBS	SARI^−/−^ (N)

### 2.7 Inflammation evaluation in the anterior chamber

The clinical signs of inflammation in the anterior chamber (eg miosis, iris hyperaemia and hypopyon) were evaluated by two experienced ophthalmologists according to the criteria modified by Li et al 24 hours after the administration of LPS.^23^

### 2.8 Histopathology

Following fixation with 4% paraformaldehyde at 4°C overnight, sagittal sections (3‐μm thick) were cut through the cornea‐optic nerve axis for pathological observation by the haematoxylin and eosin (H&E) staining assay (Beyotime). We quantified inflammatory cells in the anterior segment and posterior segment separately. Although the anterior segment includes the crystalline lens surface, the peripheral vitreous and all structures in the anterior chamber, we only choose the ciliary body and vitreous cavity to count because of the abundant collections of infiltrating inflammatory cells. A similar, we show the posterior hyaloids as key positions of the posterior segment. The numbers of inflammatory cells in the anterior segment and vitreous were counted on 5 sections per eye to provide an average cell count for quantification.

### 2.9 Statistical analyses

Data were presented as mean and Standard deviation (SD) at least 3 independent experiments. Intergroup comparisons were made by one‐way analysis of variance (ANOVA) testing, followed by *Tukey's test* for parametric data. *Kruskal‐Wallis test,* followed by *Mann‐Whitney test,* was performed for non‐parametric data between 2 groups. Statistical differences are indicated at *P*‐value < .05. Statistical tests were carried out using GraphPad Prism version 5.0 (GraphPad software).

## 3 RESULTS

### 3.1 SARI protected the leakage of CNV in mice

**Figure 1:**
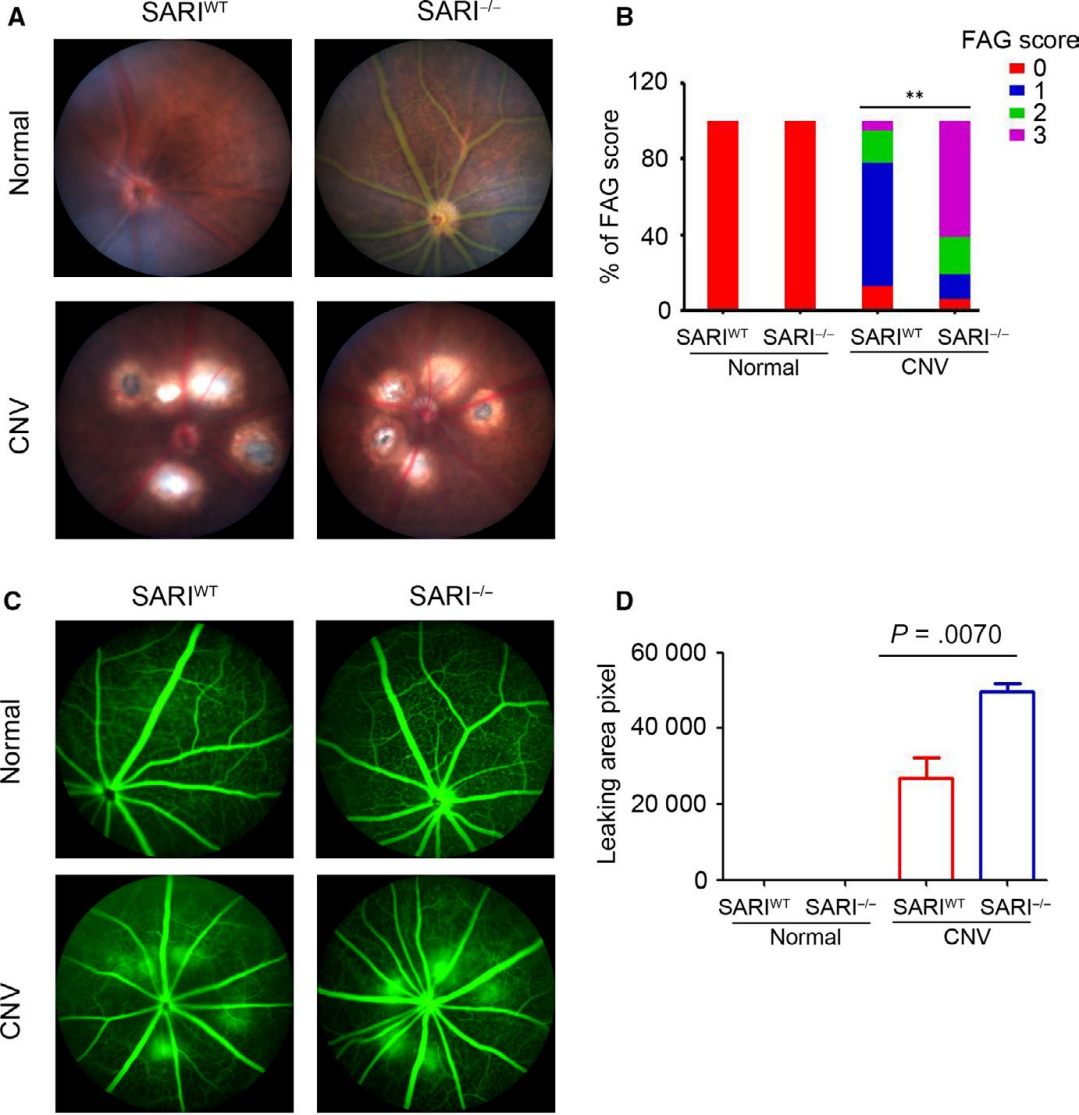
Fundus and fluorescence imaging of laser‐induced choroidal neovascularization (CNV) in mice. A, Representative images of CNV lesions in mice eyes were detected by fundoscopy. White arrows indicated the lesions of CNV. B, CNV lesions from fluorescein angiography were analysed at days 7 after treatment, and data are presented as percentage of fluorescein intensity scores (n = 42 from 8 to 10 eyes, **P < .01). Red indicates no staining (Score 0), blue indicates staining (Score 1), green indicates moderate leakage (Score 2), and purple indicates heavy leakage (Score 3). C, Representative fluorescence angiography images from each group mice, showing differences in leakage area. D, CNV areas (Hyperfluorescent leakage surrounding the laser spots) of WT group and KO group are 31 000.5 ± 11 378.1 and 59 961.5 ± 4295.1 pixel, respectively (*P* < .05), indicating a significant decline in WT group

To investigate the potential role of SARI in ocular angiogenesis, a CNV model was established in SARI^WT^ and SARI^−/−^ mice. Leakage was evaluated in vivo using fundoscopy and fluorescein angiography at 7 days after laser photocoagulation. As shown in Figure 1A, there was no scar tissue formation in the choroid of the control group. Fundus images from the CNV group showed scarring in the laser damaged spot. The scarring in the SARI^WT ^group appeared less marked at the site of injury. Representative CNV lesions were identified by fundus fluorescein angiography. Normal groups from both the wild‐ type and knockout genotype were unaffected whereas the CNV group showed significantly leakage compared to both controls. Furthermore, the SARI^−/−^ CNV group showed a similar increasing hyper fluorescence change (Figure 1C). To confirm the protective potential of SARI, the size of the leakage was graded and measured among the different genotype groups. When compared to the SARI^WT^ group, the SARI^−/−^ group showed increased area of neovascularization and leakage (Table 3). SARI deficiency was associated with decreased number of spots with weak dye staining (score 0 or 1) and increased number of spots with strong staining (score 2 or 3) (Figure 1B). Animals in the KO group also had a significantly larger leakage area of CNV than that in WT group (Figure 1D). The results suggest that SARI may play a protective role in CNV.

**Table 3:** The distribution of CNV lesion score in SARI^WT^ or SARI^−/−^ mice eyes after laser photocoagulation

Genotype	CNV lesion score			
	0	1	2	3
SARI^WT^	13%	65%	17%	5%
SARI^−/−^	6%	13%	20%	61%

### 3.2 SARI alleviates the severity of CNV in mice

**Figure 2:**
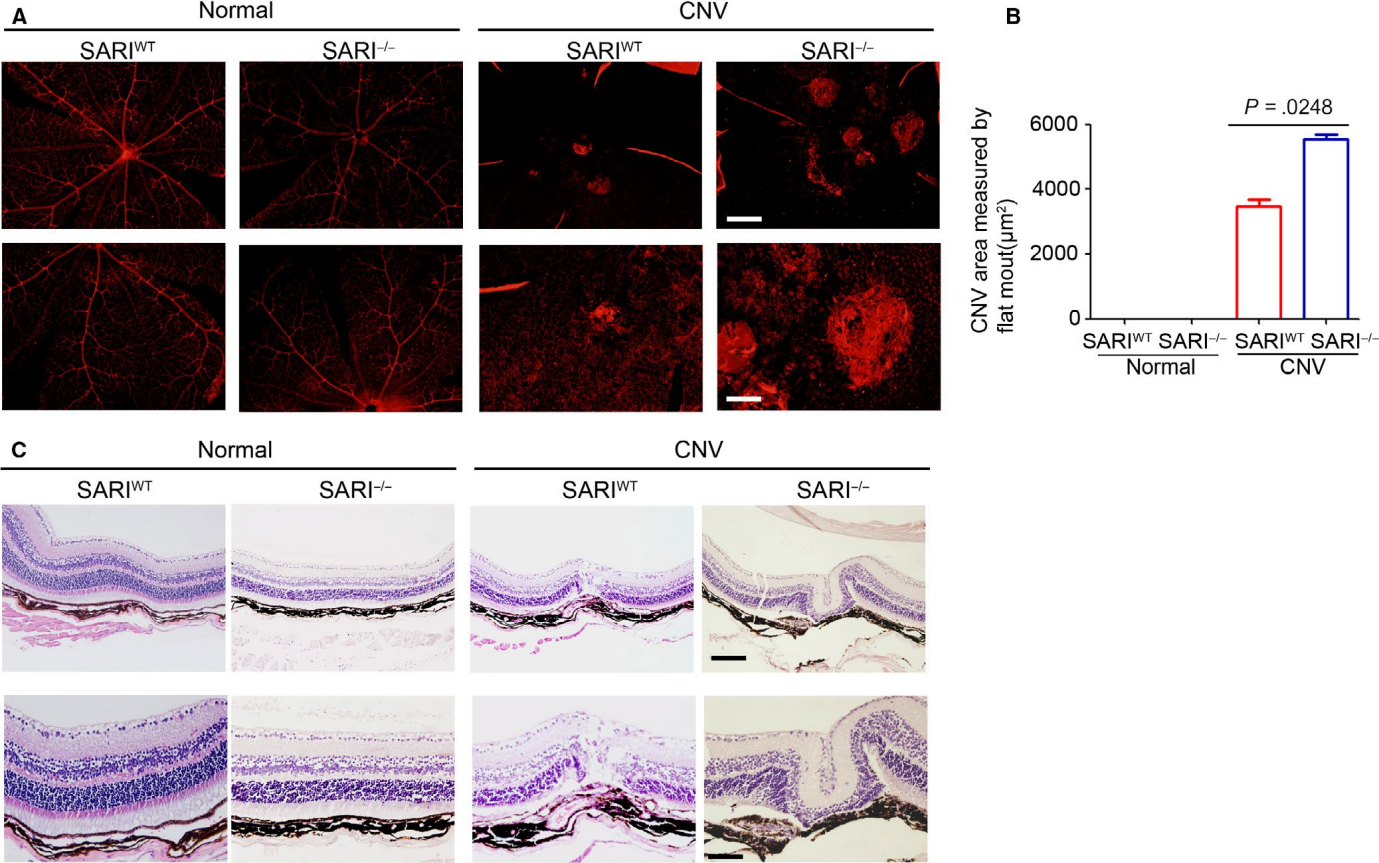
SARI inhibits CNV formation. A, Microphotographs of CNV lesion on choroidal flat mounts. Small retinal vessels, avascular areas and neovascular tufts are all shown. The choroid neovascularization was indicated by dashed circles. Scale bar = 200 μm. The lower images are inset of the upper pictures (Scale bar = 100 μm). B, Quantification of CNV 14 d after laser. C, HE stains images of CNV. RPE: retinal pigment epithelium; OS: outer segment; IS: inner segment; ONL: outer nuclear layer; OPL: outer plexiform layer; INL: inner nuclear layer; IPL: inner plexiform layer; GC: ganglion cell layer. Scale bar = 200 μm. The lower images are inset of the upper pictures (Scale bar = 100 μm)

To quantify the degree of CNV formation, we evaluated the extent of vascularization in choroidal flat ‐mounts stained against IB4. Small vessels were fully visible with fluorescence in normal groups, and the IB4‐labelled CNV outgrowths were observed in the CNV group. Such findings seem more prominent in KO mice compared to wild‐type mice (Figure 2A). The percentage of choroidal neovascularization in the control group and the CNV group was also compared to explore the function of SARI in CNV. KO mice (the CNV lesion area: 5531 ± 145.8 μm^2^, n = 30) showed a significantly expanded CNV volume compared to WT mice (the CNV lesion area: 3468 ± 197.3 μm^2^, n = 30) and controls (Figure 2B). On day 14 after laser injury, histopathology analysis showed normal mice retinal and choroid structure in the control group. Scar‐like choroidal tissue beneath the damaged retina at the site of laser injury was observed in both KO and WT CNV group. Furthermore, hyperblastosis, inflammatory cells infiltration, retinal vasculitis and folding of retina, and photoreceptor damage were more obvious in KO mice (Figure 2C). Thus, our results suggest that SARI attenuated the severity of experimental CNV.

### 3.3 SARI inhibits the expression of ocular VEGF during CNV

**Figure 3:**
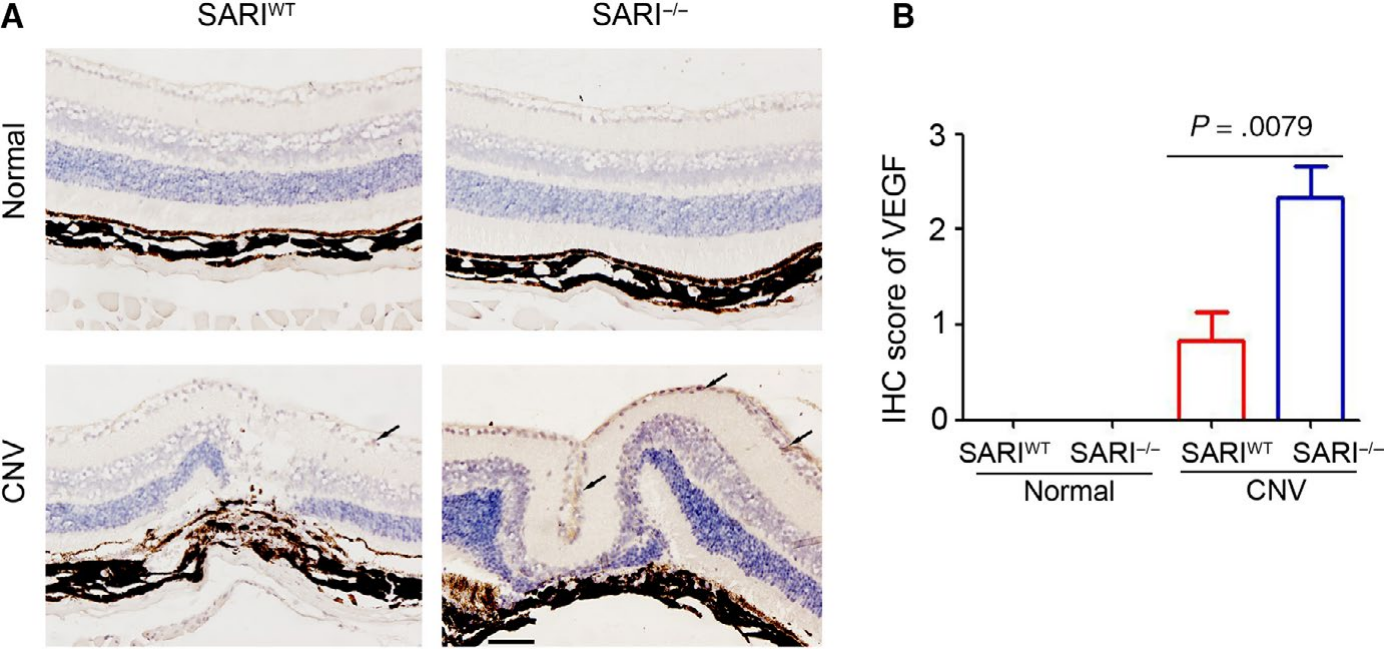
The development of laser‐induced choroidal neovascularization (CNV) is inhibited by SARI. A, Immunohistochemistry suggested that the growth of vascular endothelial growth factor (VEGF)‐labelled neovascularization in the laser‐irradiated tissue was impaired by SARI. Black arrows indicated the VEGF‐positive cells. Scale bar = 100 μm. B, Statistical analysis of neovascular lesion thickness from each group based on the immunohistochemistry (IHC) score

Many growth factors are involved in different stages of CNV.^24^ Among these growth factors, VEGF is the most important.^25^ A previous study by us has demonstrated the inhibition role of SARI on VEGF expression in colon cancer cells.^15^ To further determine the underlying mechanism of SARI attenuating the severity of CNV, the choroidal tissues were collected for VEGF staining. As shown in Figure 3A,B few VEGF‐positive cells were observed in the SARI^WT^ and SARI^−/−^ mice of normal group. Laser‐induced VEGF expression was presented in both SARI^WT^ and SARI^−/−^ mice, but more VEGF‐positive cells were observed in the choroidal tissue of SARI^−/−^ mice, compared with SARI^WT^ mice (Figure 3A,B). Collectively, the results demonstrated the inhibition role of SARI in ocular VEGF expression.

### 3.4 EIU is exacerbated in SARI‐deficient mice

**Figure 4:**
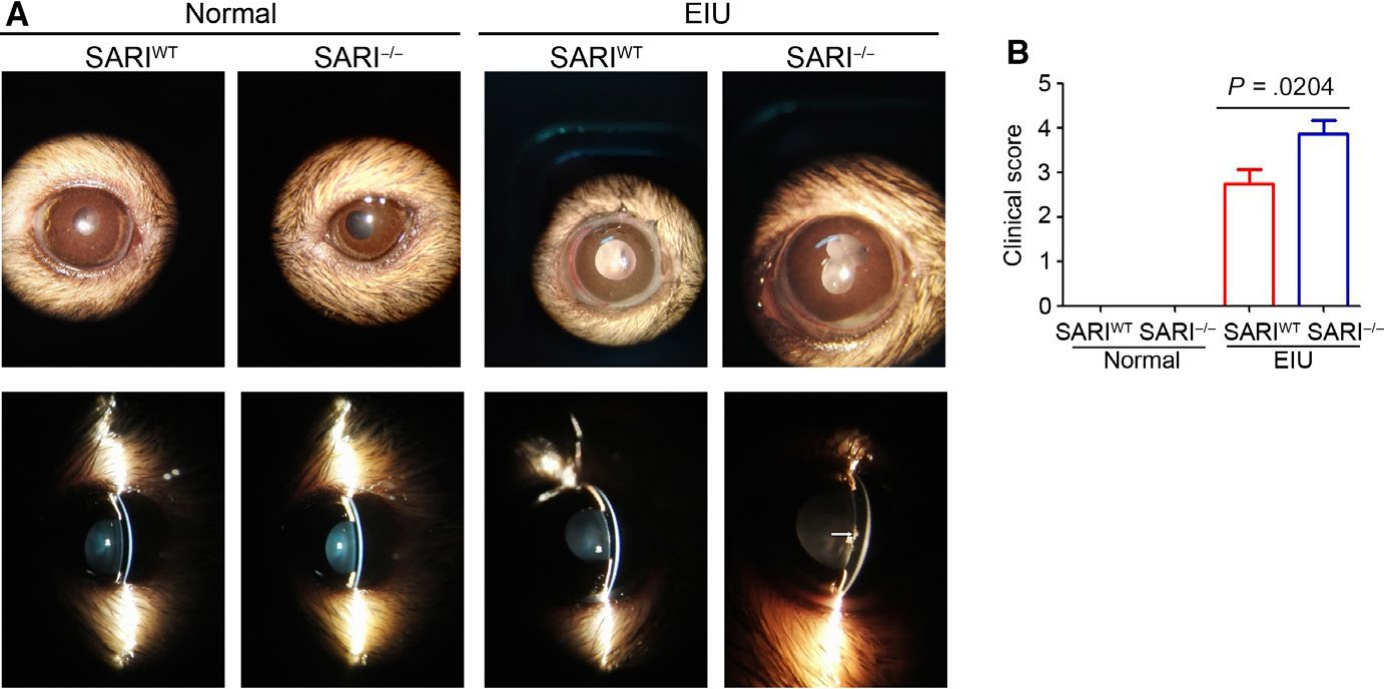
Loss of SARI exacerbates EIU. A, Gross morphological examination of eyes at 24 h after induction of EIU. The inflammatory response including iris hyperaemia, synechia and exudation is consistent with typical EIU. White arrowheads indicate the presence of fibrinoid exudation in the pupillary area, with intense flare in the anterior chamber. B, Clinical scores after LPS/PBS administration with the modified criteria. See section ‘Materials and methods’ for more details

To investigate whether SARI plays a role in regulating inflammatory cells that mediate anterior uveitis, we induced EIU in 8‐week‐old SARI^WT^ or SARI^−/−^ mice. Inflammation in the anterior chamber was assessed by a slit‐lamp biomicroscope 15 minutes before the 24th hour from intravitreal injection of LPS/PBS. In contrast to the no response of the PBS control mice, obvious inflammatory reaction with obscured anterior chamber was observed in both the SARI^WT^ group and the SARI^−/−^ group (Figure 4A). However, the inflammatory response was more severe in the KO + LPS group, as evidenced by exudation into the anterior chamber (Figure 4A). The mice in the SARI^−/−^ group had significant higher clinical score (mean, ~3.875) than that in the SARI^WT^ group (mean, ~2.750) (*P* < .05, Figure 4B). Some mice in the SARI^−/−^ group even had hypopyon. There was no inflammation seen in the SARI^−/−^ normal group or the SARI^−/−^ normal group (Figure 4B). As indicated by these results, SARI deficiency resulted in the extensive inflammation during EIU.

### 3.5 Loss of SARI increased in filtrating inflammatory cells

**Figure 5:**
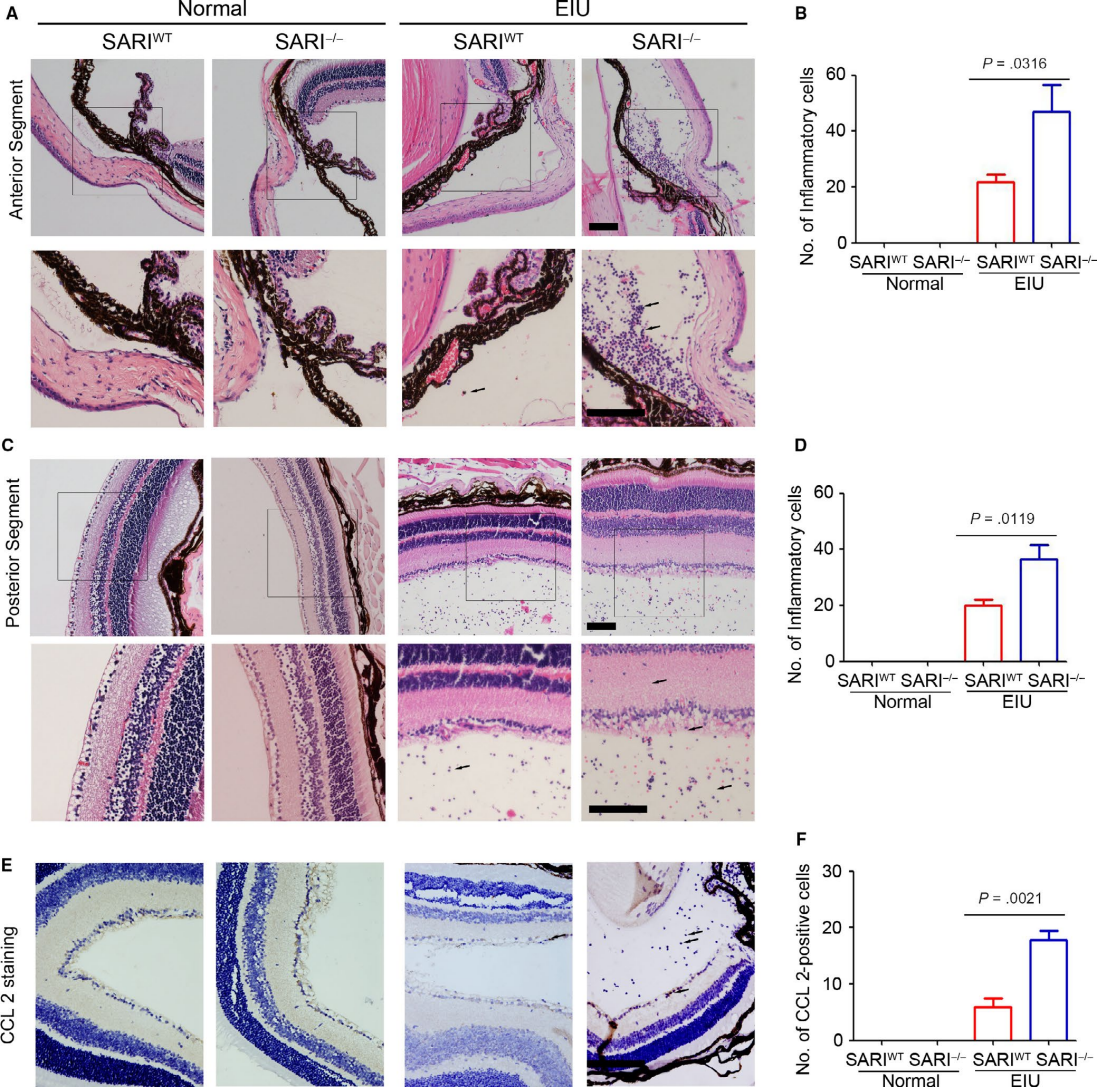
Histological evaluation from haematoxylin‐and‐eosin–stained paraffin sections at 24 h after challenge with LPS/PBS. Massive infiltration of inflammatory cells including monocytes/macrophages and neutrophils, intensive retinal vasculitis, folding of retina, and photoreceptor damage was observed in EIU group (A & C). Inflammatory cells were not observed in control groups. Black arrows indicated the inflammatory cells. Scale bar = 200 μm. Lower panel is enlarged image of upper one. Scale bar = 200 μm. B, Quantification of the percentage of inflammatory cells in the anterior segments of all groups (n = 6). D, Quantification of the percentage of inflammatory cells in the anterior segments of all groups (n = 6). E, Immunohistochemistry suggested that the expression of CCL 2 in extravascular uveal tissue was inhibited by SARI. Black arrows indicated the CCL 2‐positive cells. Scale bar = 200 μm. F, Statistical analysis of CCL 2‐positive cells in each frame of extravascular uveal tissue (n = 6)

In line with clinical observations, histological examination of the eyes 24 hours post‐injection revealed many inflammatory cells. Neutrophils and monocytes/macrophages were seen to infiltrate extravascular uveal tissue in LPS‐treated eyes and reached all the structures of interest (ciliary body, anterior, posterior and vitreous chambers, and retina). SARI deficiency caused significant increases of this effect.^26^ Conversely, the eyes in all PBS‐injected mice showed very minimal inflammatory changes with an absence of inflammatory cell infiltration in the anterior chamber and posterior segment (Figure 5A & C). Inflammatory cells did not appear to infiltrate the lens or the cornea in any group. As mentioned above, absence of SARI led to a significant increase in the inflammatory cells in the anterior chamber (Figure 5A). The EIU‐SARI^−/−^group had a 115.4% growth of infiltrating inflammatory cells in the anterior segment compared to the EIU‐SARI^WT^ group (Figure 5B). Likewise, a significant growth (82.5%) was also noted in the retina and vitreous chamber in EIU‐SARI^−/−^group vs. EIU‐SARI^WT^ (Figure 5D). According to the regulation of CCL 2‐positive inflammatory cells infiltration in colon by SARI,^26^ we further determined the expression of CCL 2 in extravascular uveal tissue. Our results suggested that SARI deficiency promoted the expression of CCL 2 in EIU model (Figure 5E & F), which indicated that more CCL 2‐positive cells infiltrated the extravascular uveal tissue. These results seem to imply that the loss of SARI led to more serious recruitment of the CCL 2‐positive inflammatory cells during EIU.

## 4 DISCUSSION

Our study demonstrated two effects of SARI on two common ocular conditions. First, SARI inhibited the spread of CNV, as shown by our high‐resolution angiography study. Second, inflammatory cells infiltration in the anterior segment and posterior segment was inhibited by SARI, which resulted in less inflammation in mice with EIU. Thus, SARI appears to play a dual function, inhibiting ocular inflammation and neovascularization. Furthermore, given that there were more neovascular and inflammation changes in SARI^−/−^ mice in the CNV and EIU models, basal SARI expression could potentially play a prognostic role in ocular diseases management. These observations contribute to the further understanding of SARI in ocular disease and provide a potential therapy target for such disease.

During CNV in wet AMD, VEGF expression is induced and plays a crucial role in mediating the process. Thus, targeting VEGF is an efficient strategy for treating wet AMD.^27^, ^28^ Currently, several neutralizing antibodies targeting VEGF, including bevacizumab, ranibizumab and aflibercept, are used for treating neovascular AMD and show good therapeutic effect clinically.^29^, ^30^, ^31^ Dual functional neutralizing antibody IBI302 targeting VEGF and complement system also showed promise as a candidate for AMD treatment.^32^ In our study, results demonstrated the protective role of SARI in laser‐induced CNV model in mice, evidenced by less leakage and severity of CNV in SARI^WT^ mice. Underlying mechanism investigations indicated that fewer VEGF‐positive cells were observed in the choroidal tissue of SARI^WT^ mice. The inhibitional role of SARI on ocular VEGF expression was consistent with our previous study, which showed the anti‐angiogenesis role and VEGF expression of SARI in colon cancer cells and tumours.^15^

Inflammation is the major cause of uveitis. In ocular inflammation, macrophages are located mostly in the surrounding inflammatory tissue and are essential for the vascularization and damage of the inflammatory tissue.^33^ Thus, targeting macrophage is an efficient treatment strategy for inflammatory ocular disease,^34^ and for ocular diseases involving neovascularization.^35^ According to the chemotaxis of CCL 2 on macrophage infiltration, CCL 2 inhibitor shows a good therapeutic effect on ocular‐related diseases.^36^ In a previous study, we have demonstrated the inhibitory role of SARI in CCL 2 expression in epithelial colon cells through promoting the degradation of STAT1 under colitis condition.^26^ SARI inhibited the infiltration of macrophage into colon tissues and colitis development in mice.^26^ In the present study, we also confirmed the protective role of SARI in EIU, which may contribute to the inhibition of CCR 2‐positive inflammatory cells infiltration in the anterior segment and posterior segment.

In summary, the present study demonstrated the dual functional role of SARI in ocular disease, inhibiting ocular inflammation and neovascularization, which suggests that SARI could be a potential therapy target for certain ocular diseases. However, the present study only investigated the functional role and underlying mechanism of SARI in regulating wet AMD and EIU in SARI^WT^ and SARI^−/−^ mice. Further studies are needed to gain a deeper understanding of the SARI regulatory pathways in order to identify potential targets for intervention.

## CONFLICT OF INTEREST

The authors declare that they have no potential conflict of interest.

## AUTHOR CONTRIBUTIONS

Wenqiu Zhang, Lei Dai, Xun Li and Yiming Li were involved in acquisition and analysis of the data. Hongxin Deng and Longqian Liu were involved in the study concept and design and obtained funding. Maurice Keng Hung Yap was involved in critical revision of the manuscript for important intellectual content. Wenqiu Zhang and Lei Dai were involved in writing the manuscript.
